# Enhancing the Photocatalytic Degradation of Methylene Blue with Graphene Oxide-Encapsulated g-C_3_N_4_/ZnO Ternary Composites

**DOI:** 10.1021/acsomega.3c10172

**Published:** 2024-03-26

**Authors:** Muhammad Hassan Shakoor, Muhammad Bilal Shakoor, Asim Jilani, Toheed Ahmed, Muhammad Rizwan, Mohsin Raza Dustgeer, Javed Iqbal, Muhammad Zahid, Jean Wan Hong Yong

**Affiliations:** † Department of Chemistry, Riphah International University, Faisalabad Campus, Faisalabad 38000, Pakistan; ‡ College of Earth & Environmental Sciences, University of the Punjab, Lahore 54590, Pakistan; § Center of Nanotechnology, King Abdulaziz University, 21589 Jeddah, Saudi Arabia; ∥ Department of Environmental Sciences and Engineering, Government College University Faisalabad, Faisalabad 38000, Pakistan; ⊥ Department of Chemistry, University of Agriculture, Faisalabad 38000, Pakistan; # Department of Biosystems and Technology, Swedish University of Agricultural Sciences, 23456 Alnarp, Sweden

## Abstract

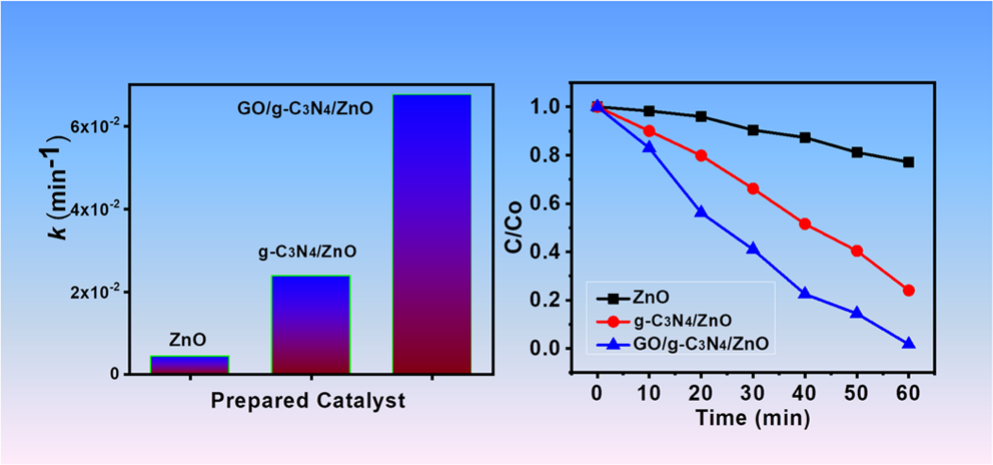

Methylene blue (MB) is a toxic contaminant present in wastewater. Here, we prepared various composites of graphene oxide (GO) with graphitic carbon nitride (g-C_3_N_4_) and zinc oxide (ZnO) for the degradation of MB. In comparison to ZnO (22.9%) and g-C_3_N_4_/ZnO (76.0%), the ternary composites of GO/g-C_3_N_4_/ZnO showed 90% photocatalytic degradation of MB under a light source after 60 min. The experimental setup and parameters were varied to examine the process and effectiveness of MB degradation. Based on the results of the experiments, a proposed photocatalytic degradation process that explains the roles of GO, ZnO, and g-C_3_N_4_ in improving the photocatalytic efficacy of newly prepared GO/g-C_3_N_4_/ZnO was explored. Notably, the g-C_3_N_4_/ZnO nanocomposite’s surface was uniformly covered with ZnO nanorods. The images of the samples clearly demonstrated the porous nature of GO/g-C_3_N_4_/ZnO photocatalysts, and even after being mixed with GO, the g-C_3_N_4_/ZnO composite retained the layered structure of the original material. The catalyst’s porous structure plausibly enhanced the degradation of the contaminants. The high-clarity production of g-C_3_N_4_ and the effectiveness of the synthesis protocol were later validated by the absence of any trace contamination in the energy-dispersive X-ray spectroscopy (EDS) results. The composition of the ZnO elements and their spectra were revealed by the EDS results of the prepared ZnO nanorods, g-C_3_N_4_/ZnO, and GO/g-C_3_N_4/_ZnO. The outcomes indicated that the nanocomposites were highly uncontaminated and contained all necessary elements to facilitate the transformative process. The results of this experiment could be applied at a large scale, thus proving the effectiveness of photocatalysts for the removal of dyes.

## 1 Introduction

Water pollution is the contamination of natural water resources, such as lakes, rivers, oceans, and groundwater, with substances that are hazardous to living organisms and the ecosystems. Methylene blue (MB) is an aromatic compound having a heterocyclic structure. It is frequently used dye in paper and textile, wool, and silk industry.^1^ If ingested or comes into direct contact with the skin surface, MB is extremely dangerous. The discharge of partially or untreated MB dye-loaded wastewater from any industrial production may pose a number of health risks.^2^ In humans, for example, MB dye can cause jaundice, cyanosis, tissue necrosis, vomiting, higher heart rate, and cancers of different types.^3^ Furthermore, the existence of MB has evolved into a massive issue for plants, causing growth inhibition, pigment minimization, and a reduction in the microalgae protein content. As a matter of fact, before releasing MB-polluted wastewater into freshwater environments, this poisonous dye should be removed.^4^

Contamination from water can be reduced using the energy of light via a process called photocatalysis. A photocatalyst is a substance that can absorb light energy and utilize it to stimulate chemical reactions which is the main part of this process.^5^ One benefit of treatment of wastewater by photocatalysis is that a variety of pollutants, including those that are challenging to remove by other methods, can be broken down using this approach.^6,7^ The quantity of impurities released into the surroundings can be minimized by using treatment of wastewater by photocatalysis, which can be more effective and affordable than conventional chemical treatment approaches.^8^ The photocatalytic process can be applied in a variety of methods; for example, employing a photocatalytic reactor, which is an enclosed area where water is exposed to light while being in the presence of a photocatalyst, is one favored technique. Additionally, it can be used to detoxify water by destroying microorganisms.^9,10^ Materials containing at least a single dimension in the nanoscale range are called nanomaterials (typically between 1 and 100 nm).^11^ These substances have unique characteristics that make them beneficial for treating wastewater.^12,13^ Graphene and carbon nanotubes are two main examples of nanoparticles containing a high surface area-to-volume ratio. These substances are easily eliminated from water for disposal and have a significant capacity for adsorbing contaminants. Another technique for disinfecting wastewater with nanomaterials is facilitated by harnessing the catalytic processes, which is the stimulation of several chemical reactions.^14^ Metallic nanoparticles like silver and gold can stimulate the degradation of wastewater pollutants like microorganisms and organic substances.^15^ Additionally, photocatalysis,^16^ as we previously discussed, is a different method of water purification that may be applied by using semiconductor nanoparticles, including silicone dioxide, titanium dioxide, and zinc oxide as a photocatalyst, which is more effective and demands fewer doses of ultraviolet light. Overall, introducing nanomaterials to water treatment shows potential for raising the effectiveness and efficiency of existing wastewater treatment systems.^17,18^ Before bringing these materials on a commercial scale, it is crucial to carefully assess the potential effects they may have on human health and the environment and put safety precautions in position. Zinc oxide (ZnO) is a semiconductor substance that has been investigated as a photocatalyst for the decontamination of wastewater.^19^ As a photocatalyst, ZnO has several benefits, including its wide range of band gap, which enables it to absorb an extensive range of wavelengths of light, and its relatively low toxicity. Zinc oxide produces hole pairs of electrons when subjected to light, which can then be used to oxidize contaminants in water.^20^ It has been discovered that ZnO is good in oxidizing a variety of contaminants, including organic materials, microorganisms, and pathogens. Additionally, the performance of ZnO is improved by preparing ZnO nanoparticles having enlarged surface area-to-volume ratio thus improving the active sites for photocatalytic degradation of pollutants.^21^ The ZnO can also be combined with various metallic nanoparticles, which could act as cocatalysts and enhance the activity of ZnO in photocatalysis.^22^ It is important to keep in mind that ZnO has several limitations, including the fact that photocatalytic activity is only limited to visible light, which means that UV light is the main activator for the photocatalytic process. Furthermore, it has been discovered that ZnO is less persistent in acidic and basic conditions, which may restrict its application in particular wastewater treatment scenarios.^23^ However, due to its wide range of band gap, low degree of toxicity, and adaptability in a variety of applications, ZnO is a potential photocatalyst for wastewater treatment; nevertheless, more research is required to improve its performance and overcome its drawbacks.^24^

The graphitic carbon nitride (g-C_3_N_4_) is used to purify wastewater through an approach called photocatalytic oxidation, which uses the energy of light to degrade contaminants in water. Reactive oxygen species, such as hydroxyl radicals, can be produced using g-C_3_N_4_ and subsequently can be used to degrade harmful substances in water. Making g-C_3_N_4_-based composite materials is yet another method where g-C_3_N_4_ is used to remediate water.^25^ To increase the photocatalytic activity of g-C_3_N_4_, researchers have been investigating ways of merging it with other substances, such as metal nanoparticles, which may operate as cocatalysts.^26^ It was known that the g-C_3_N_4_ is effective at degrading different contaminants and volatile organic compounds in other applications, through a process of air filtration.^27^ Nevertheless, more research pertaining to g-C_3_N_4_ is needed before it can be used extensively in water treatment systems. For example, new study is required to improve the synthesis of g-C_3_N_4_ and to produce it at a lower cost on a larger scale. To facilitate large-scale implementation and with human health consideration, new research is required to evaluate the enduring stability and safety hazards of g-C_3_N_4_ in applications involving water treatment. As a powerful photocatalyst for wastewater treatment, g-C_3_N_4_ has a wide range of uses and was proven to be highly effective at removing contaminants under laboratory conditions.^28^

Graphene is considered an important 2D material for different applications such as heterogeneous catalysis, adsorption, and photocatalysis owing to its promising characteristics including outstanding thermal conductivity, higher mechanical strength, and electron mobility.^29,30^ Graphene is a highly efficient photocatalytic material due to its high surface area providing a suitable support for ZnO nanoparticles and thus improving the photocatalytic degradation of pollutants.^31,32^ Graphene oxide (GO) is a promising material for water treatment with high adsorption capacity, catalytic activity, and mechanical stability. But its scalability, cost-effectiveness, and safety require further assessment and consideration.^33^ Therefore, herein, we prepared a combination of ZnO with GO and g-C_3_N_4_ using the wet impregnation method. The role of GO and g-C_3_N_4_ in improving the photocatalytic performance of ZnO was assessed. Furthermore, the variations in surface functional groups using the XPS technique and changes in the optical and structural features of prepared nanocomposites were also determined.

## 2 Material and Methods

### 2.1 Materials

Analytical grade sulfuric acid (H_2_SO_4_) (95–98%), graphite (99%), hydrogen peroxide (H_2_O_2_) (37%), and phosphoric acid (H_3_PO_4_) (85%) were bought from Sigma-Aldrich company. Zinc acetate dihydrate (99.99%) and potassium permanganate (KMnO_4_) (97%) were obtained from Merck and Alfa Aesar, respectively. Methylene blue (95%), melamine (99%), and graphite flakes were bought from the internationally reputable chemical company (Sigma-Aldrich) and used in the tests without further processing.

### 2.2 Preparation of Graphene oxide

The most common and widely used method is Hummer’s method for making graphene oxide. Graphene oxide (GO) is also sometimes called graphitic acid. For graphene oxide preparation, 3 g of graphite and 18 g of KMnO_4_ with a 9:1 ratio of H_2_SO_4_ to H_3_PO_4_ was mixed and the final volume was made up to 400 mL. After 12 h of continuous stirring at a temperature of 50 °C, the reaction mixture was poured into 400 mL of ice by making an ice bath. After the mixture was cooled while being continuously stirred by a glass rod, it was treated with H_2_O_2_ having a quantity of 3 mL for precipitated GO. The mixture of GO was washed with hydrochloric acid (HCl) (5%), deionized water, and ethanol before filtration. Finally, GO solution was obtained and centrifuged for additional purification and graphene oxide sheets preparation.^34^

### 2.3 Preparation of Zinc Oxide

Zinc oxide (ZnO) nanoparticles were prepared by direct heating. Zinc acetate was used as a precursor to create ZnO photocatalyst. 50 mL of ethanol was combined with 3 g of zinc acetate dihydrate and vigorously stirred. The material was shifted to an aluminum crucible after 2 h of continuous stirring, where it was heated for 60 min at 400 °C in a muffle furnace to convert it into ZnO. Finally, we obtained the ZnO nanoparticles which were converted into a powdered form.^35^

### 2.4 Preparation of Graphitic Carbon Nitride (**g-C**_**3**_**N**_**4**_)

5 g of melamine was weighed at room temperature, covered with a ceramic crucible, and then placed in a muffle furnace for heating. The temperature of heating was increased at 20 °C/min and continued for 2 h. The final temperature achieved was 550 °C. The sample was kept for heating in a furnace for 5 h. After heating, the crucible was cooled and the sample was converted into a powdered form.^36^

### 2.5 Synthesis of GO/g-C_3_N_4_/ZnO

The GO/g-C_3_N_4_/ZnO was prepared by a wet impregnation method. This method was beneficial for the synthesis of GO/g-C_3_N_4_/ZnO due to its simplicity and low synthesizing cost for photocatalytic degradation. 2% weight of graphene oxide, 38% weight of g-C_3_N_4_, and 60% weight of ZnO were combined in ethanol solution. The mixture was continuously stirred for 120 min. The centrifuge technique was used to separate the ethanol from the mixture at 3500 rpm. The obtained mixture was dried in an oven for 120 min to get the final product of GO/g-C_3_N_4_/ZnO.^37^

### 2.6 Characterization of GO/*g*-C_3_N_4_/ZnO

The GO/g-C_3_N_4_/ZnO structure was examined using a Rigaku Ultima IV X-ray diffraction (XRD). Versa PobeII X-ray photoelectron spectroscopy (XPS) was used to investigate the surface characteristics and chemical composition. Surface morphology was examined using a scanning electron microscope (JEOL, JSM7600-F). The absorption spectra for the degradation of methylene blue (MB) were obtained through a spectrophotometer (DR 6000, Hach Lang).

### 2.7 Photocatalytic Degradation Test

In order to test the photocatalytic abilities of GO/g-C_3_N_4_/ZnO, 20 mg of the catalysts was added to 200 mL of 15 ppm of MB. To achieve absorption equilibrium, the solution was continuously stirred while being kept in the dark for 1 h. The solution was then exposed to visible light (100 W) for the following 60 min, with 2 mL being taken every 10 min for deterioration analysis.^38^ The degradation percentage and reaction rate constant were determined using the formulas below (eqs 1 and 2):
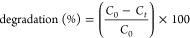
1
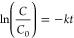
2

## 3 Results and Discussion

### 3.1 Surface Compositional Analysis

**Figure 1:**
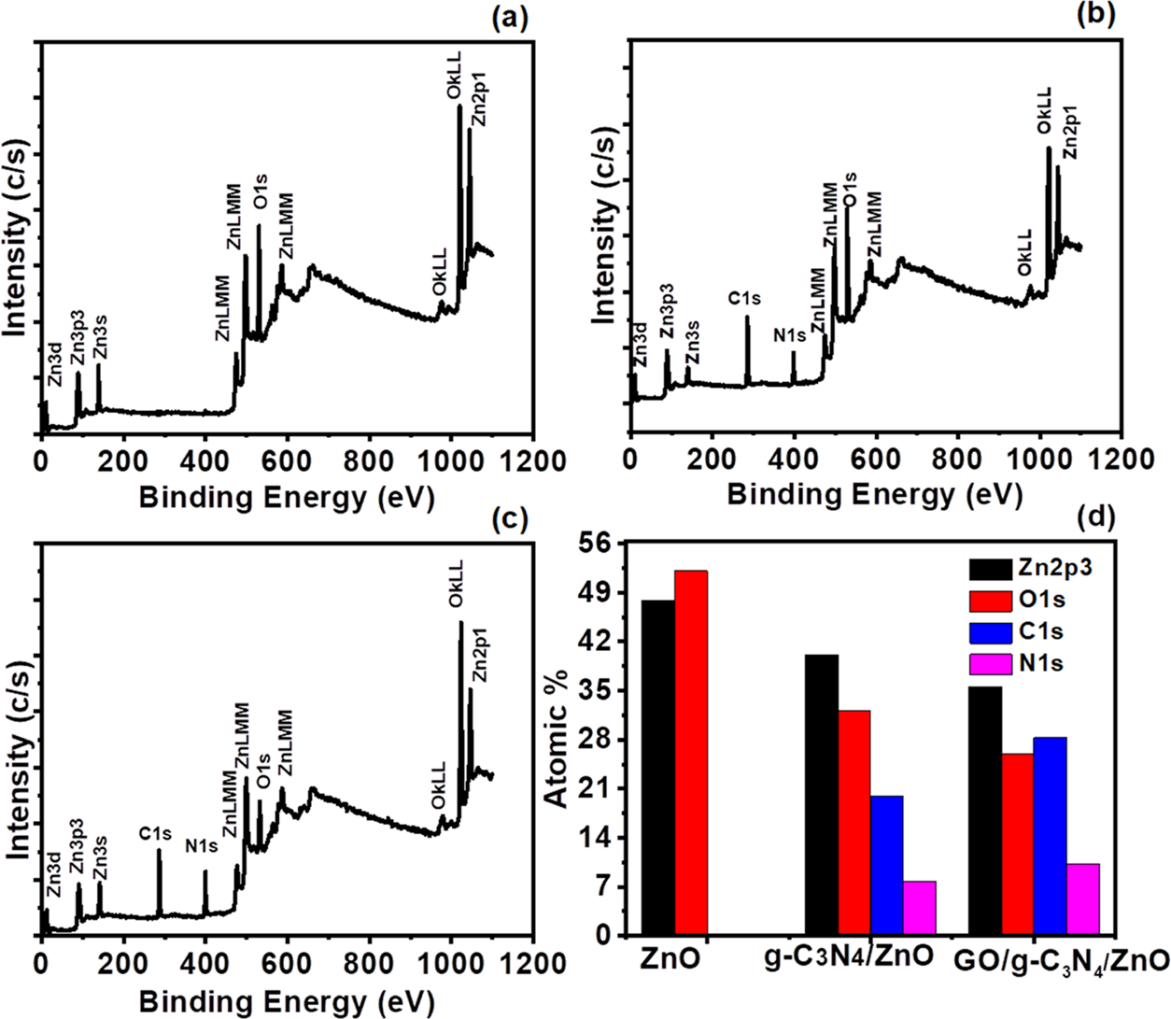
XPS spectra of ZnO (a), g-C_3_N_4_/ZnO (b), and GO/g-C_3_N_4_/ZnO (c) and atomic percentage of ZnO, g-C_3_N_4_/ZnO, and GO/g-C_3_N_4_/ZnO (d).

The O1s spectra of zinc oxide (ZnO) (Figure 1a) demonstrated the presence of only one peak at around 530 eV which was attributed to the presence of Zn–O having 50% atomic ratio. However, C 1s spectra were present in g-C_3_N_4_/ZnO as well as the N1s and O1s peaks were also observed at 290, 400, and 540 eV.^39^ These spectra showed that their atomic ratios are 20, 8, and 33% respectively. After adding graphitic carbon nitride (g-C_3_N_4_) to ZnO, a small shift was observed in O1s spectra as compared to ZnO only. The atomic percentage of C=O=C decreased from 50 to 33%.^40^ The C1s, N1s, and O1s spectra in GO/g-C_3_N_4_/ZnO comparatively also observed around the peaks of 290, 400, and 540 eV, which are attributed to the atomic ratios of related elements, that is, 26, 10, and 24%, respectively.^40^

The Zn2p3 spectrum of GO/g-C_3_N_4_/ZnO (depicted in Figure S1a) exhibits three discernible peaks at approximately 1019, 1022, and 1025 eV. The peak at 1019 eV is ascribed to Zn^2+^, while the peaks at 1022 and 1025 eV are indicative of zinc interacting with oxygen (Zn–O) and hydroxyl groups, respectively.^41^ Analyzing the O1s spectra of ZnO (as shown in Figure S1b) reveals three distinctive peaks at approximately 529, 531, and 533 eV, corresponding to metal interaction (O metal), oxygen vacancies (O_V_), and hydroxyl interaction (OH), respectively.^41^ The existence of oxygen vacancies and hydroxyl interactions creates binding sites that facilitate the capture of dye molecules, effectively reducing the charge recombination ratio. This phenomenon contributes to the augmented photocatalytic degradation of ZnO-based composites.^42^

### 3.2 XRD

**Figure 2:**
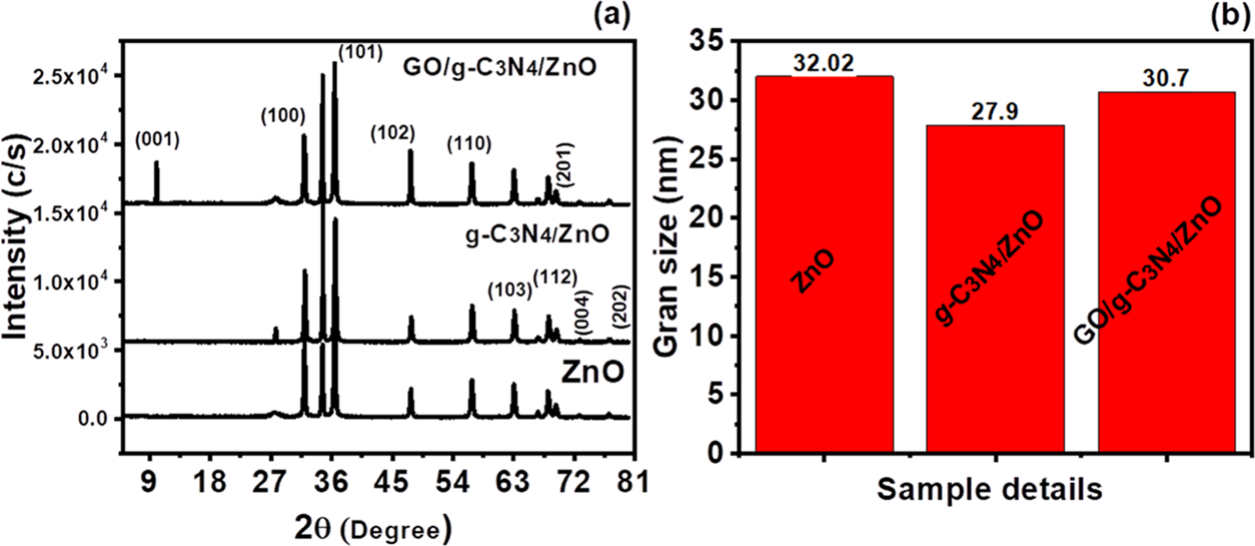
(a) Diffraction patterns of ZnO, g-C_3_N_4_/ZnO, and GO/g-C_3_N_4_/ZnO. (b) Grain size of ZnO, g-C_3_N_4_/ZnO, and GO/g-C_3_N_4_/ZnO.

X-ray diffraction (XRD) analysis was employed to investigate the structural properties and crystallinity of pristine g-C_3_N_4_ and ZnO, as well as their composite materials (GO/g-C_3_N_4_/ZnO). The spectra of ZnO revealed a prominent peak at 2θ = 36.40°, which was attributed to the diffraction plane (101) (Figure 2). Additionally, there were less intense peaks observed at 2θ = 32.02, 34.73, 48.50, 55.75, 63.09, 66.50, 68.80, 69.30, and 72.70°, attributed to planes (100), (001), (102), (110), (103), (201), (200), (112), and (004), respectively. The ZnO diffraction pattern alignment with the JCPD 01-073-6865 card indicated that ZnO nanorods possessed a zincite structure. In g-C_3_N_4_/ZnO, the XRD spectrum showed that distinct ZnO peaks were observed, in conjunction with a weak g-C_3_N_4_ peak (Figure 2a). The ternary composite (GO/g-C_3_N_4_/ZnO) exhibited identical peak patterns of diffraction. However, the GO component did not exhibit sharp peaks; instead, it displayed a little dip which was overshadowed due to the occurrence of strong peaks. Nevertheless, a weak g-C_3_N_4_ peak was discernible in the spectrum of ternary nanocomposite, the same as observed in g-C_3_N_4_/ZnO composites (Figure 2a).^37^ The existence of ZnO and g-C_3_N_4_/ZnO diffraction peak patterns in the prepared nanocomposites confirmed the preparation of ternary composites, which exhibited crystalline properties. The mean grain size of individual catalysts and their composites was determined using the Scherrer equation (eq 3)^43^
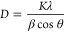
3

The average crystalline size (*D*) was calculated using the Scherrer equation, where β shows the full width at half-maximum of the diffraction peak patterns, while λ and K represent wavelength and constant, respectively. The average grain size of ZnO was determined to be 32.02 nm (Figure 2b).^44^ Furthermore, the influence of ZnO lattice dislocation can be calculated based on the following relationship (eq 4):^37^
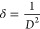
4

The results showed that the dislocation densities for ZnO, g-C_3_N_4_/ZnO, and GO/g-C3N4/ZnO were calculated to be 8.46 × 10^–3^, 2.69 × 10^–4^, and 2.96 × 10^–4^ nm, respectively. Changes in the dislocation density might influence the lattice strain of prepared ternary composite materials. Moreover, the lattice strain can be calculated using the following relationship (eq 5):^45^
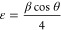
5

The estimated lattice strain values for ZnO, g-C_3_N_4/_ZnO, and GO/g-C_3_N_4_/ZnO were 1.03 × 10^–3^, 1.41 × 10^–3^, and 1.25 × 10^–3^ nm, respectively.^46^

### 3.3 SEM

**Figure 3:**
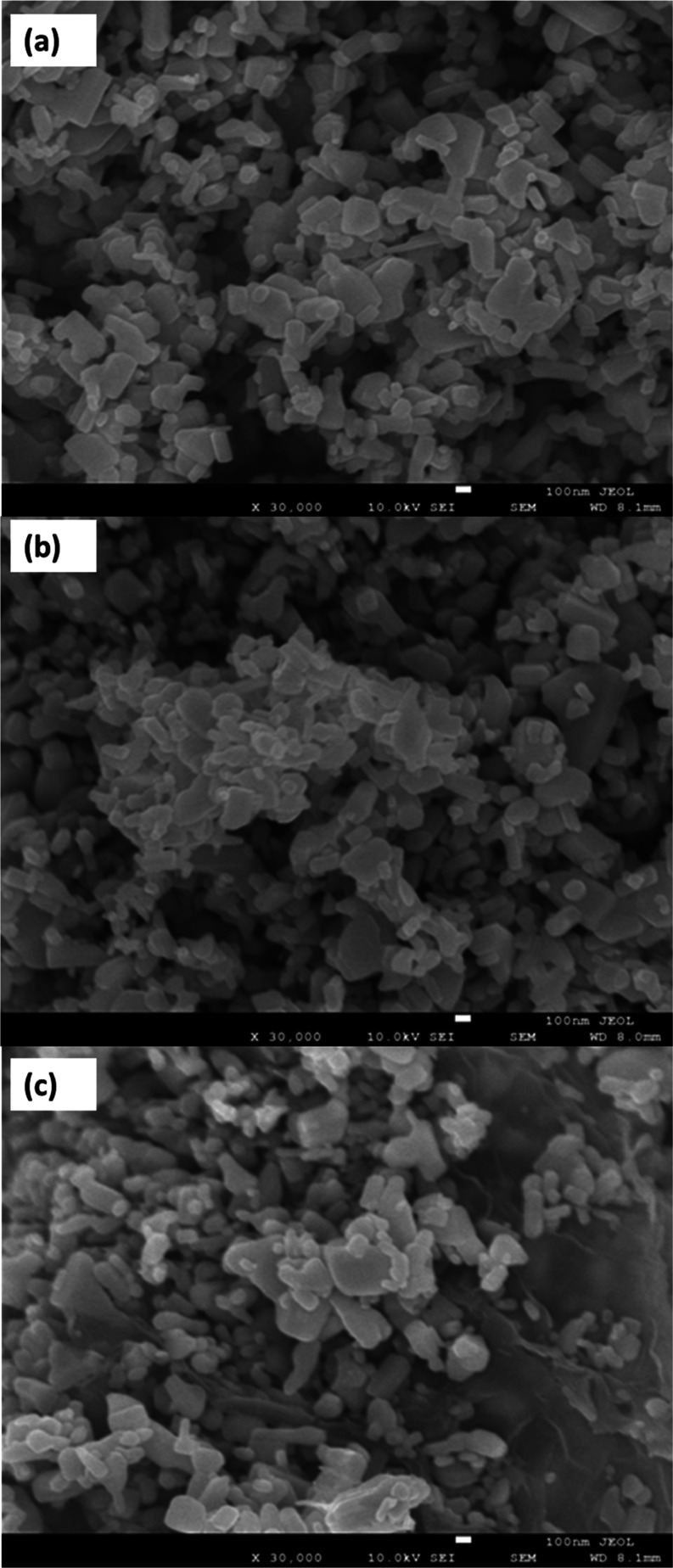
FESEM images of ZnO (a), g-C_3_N_4_/ZnO (b), and GO/g-C_3_N_4_/ZnO (c).

The surface characterization of the sample was conducted by using field emission scanning electron microscopy (FESEM). The images analyzed by FESEM, which were taken at ×30k magnification, are presented in Figure 3. Figure 3 revealed the rodlike structures within the composition of ZnO. In Figure 3b, a binary composite (g-C_3_N_4_/ZnO) is shown and showcasing ZnO nanorod structures alongside g-C_3_N_4_ particles. Figure 3c highlighted the GO sheets in addition to ZnO nanorods and also the unusually spherical-based g-C_3_N_4_ structures. The ZnO, which is the main material, maintained its rodlike distinct structure in both prepared nanocomposites, as illustrated in Figure 3a–c.^47^

### 3.4 EDS Spectra

**Figure 4:**
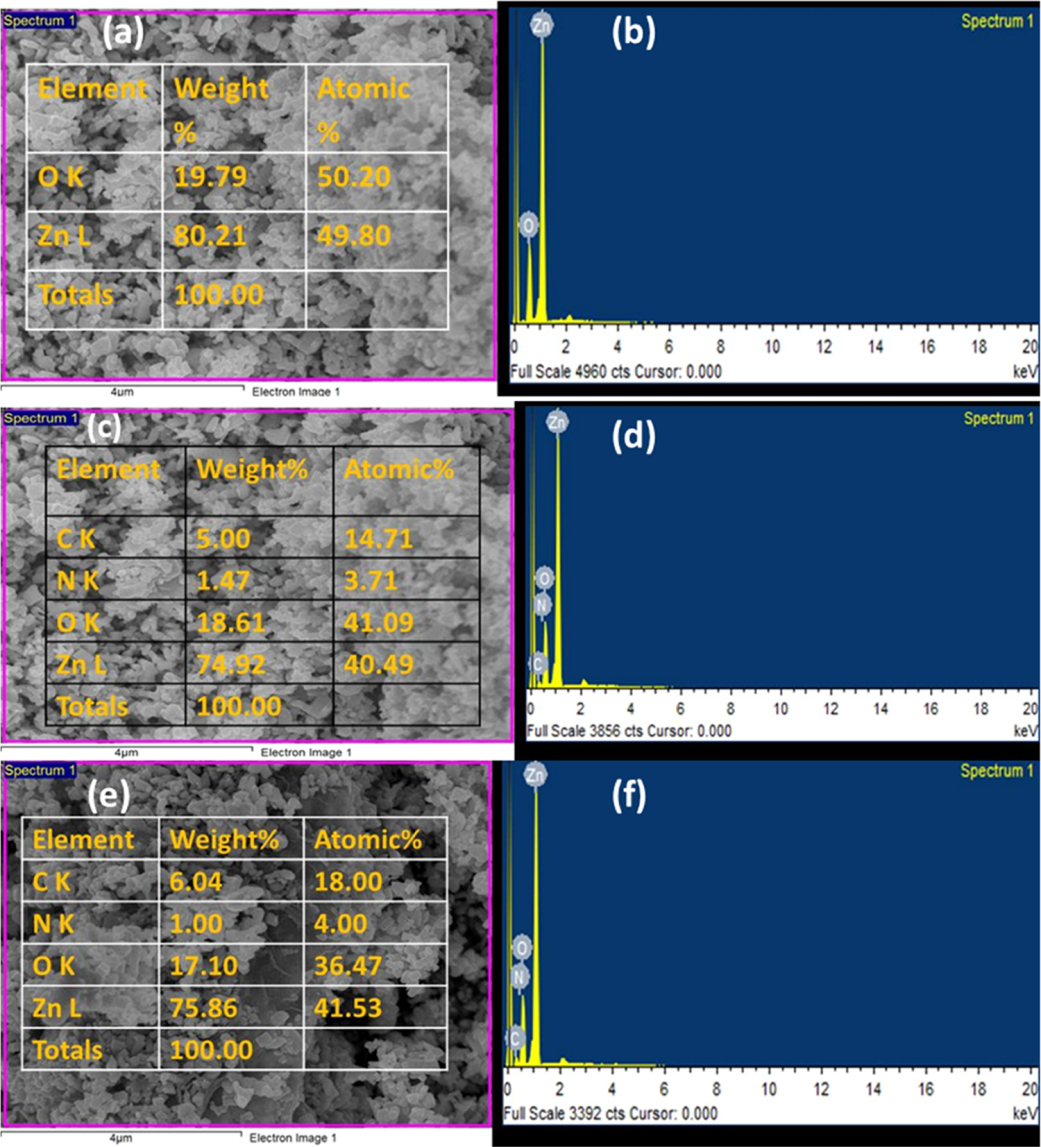
EDS compositional analyses and spectra of ZnO (a, b), g-C_3_N_4_/ZnO (c, d), and GO/g-C_3_N_4_/ZnO (e, f).

All prepared samples were analyzed for elemental composition using energy-dispersive X-ray spectroscopy (EDS), and data are depicted in Figure 4. Figure 4a,b provided the elemental composition and EDS spectra of the newly prepared ZnO nanorods. Likewise, Figure 4c–f revealed the EDS outcomes for the binary (g-C_3_N_4_/ZnO) and ternary (GO/g-C_3_N_4_/ZnO) nanocomposites. The outcomes provided good evidence that the prepared nanocomposites exhibited exceptional purity and encompassing all of the necessary elements.^48^

The elemental mapping analysis of the ternary composite, GO/g-C_3_N_4_/ZnO, through EDS revealed a uniform distribution of constituent elements across the surface area. The compounded image in Figure S2a and individual elemental distributions in Figure S2b–e illustrate the even dispersion of carbon, nitrogen, oxygen, and zinc. This uniform distribution is a noteworthy observation, emphasizing the homogeneity of the ternary composite.

### 3.5 Photocatalytic Degradation Study

**Figure 5:**
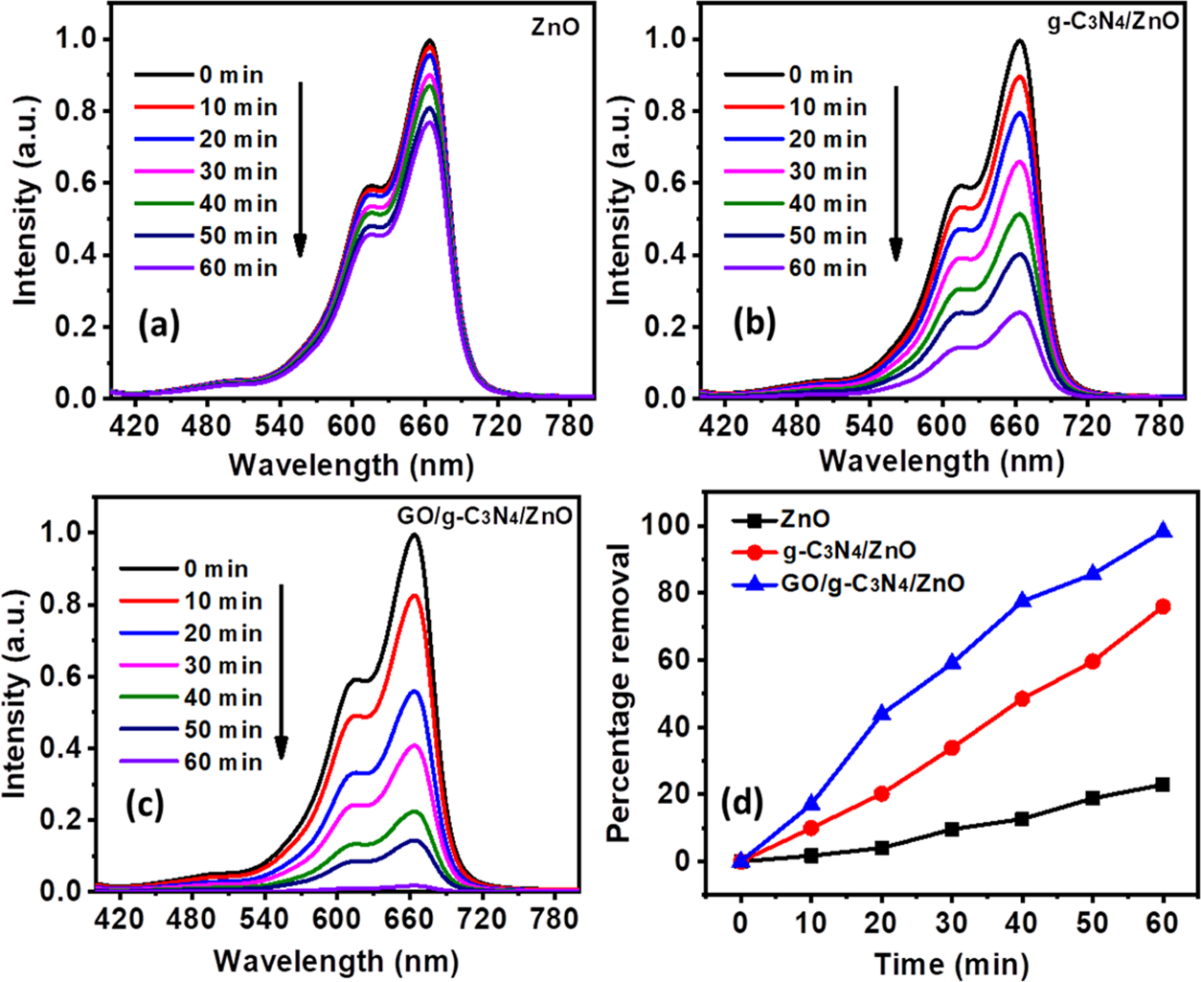
Photocatalytic degradation of ZnO (a), g-C_3_N_4_/ZnO (b), and GO/g-C_3_N_4_/ZnO (c), and percentage removal (d).

The putative pollutant methylene blue dye was used to test the photocatalytic activity of ZnO, g-C_3_N_4_/ZnO, and GO/g-C_3_N_4_/ZnO in a batch reactor. A novel process involved mixing 50 mL of 200 mg/L dye and 0.1 g of the produced catalyst, agitating the mixture for 30 min in the dark. The outcomes were highly reproducible to within 5%. Figure 5a showed the results of the evaluation of ZnO’s capacity to photocatalytically degrade MB. After 60 min, the absorbance peak reduced significantly for ZnO, g-C_3_N_4_/ZnO, and GO/g-C_3_N_4_/ZnO (Figure 5a–c). ZnO displayed the MB degradation of 22.88%. ZnO has a lower rate of degradation, which is attributed to both its large band gap, as shown in Figure 5a, which restricted light absorption, and accelerated rate of electron–hole pair recombination during the process of conduction.^49^ g-C_3_N_4_/ZnO demonstrated a 75.96% degradation of methylene blue; this increased MB degradation percentage was attributed to the narrower band gap,^50^ which possibly enabled more visible light absorption, as shown in Figure 5b. In addition, it was discovered that combining ZnO, g-C_3_N_4_, and GO further improved MB degradation and leading to 90% degradation, as revealed by Figure 5c,d.

**Figure 6:**
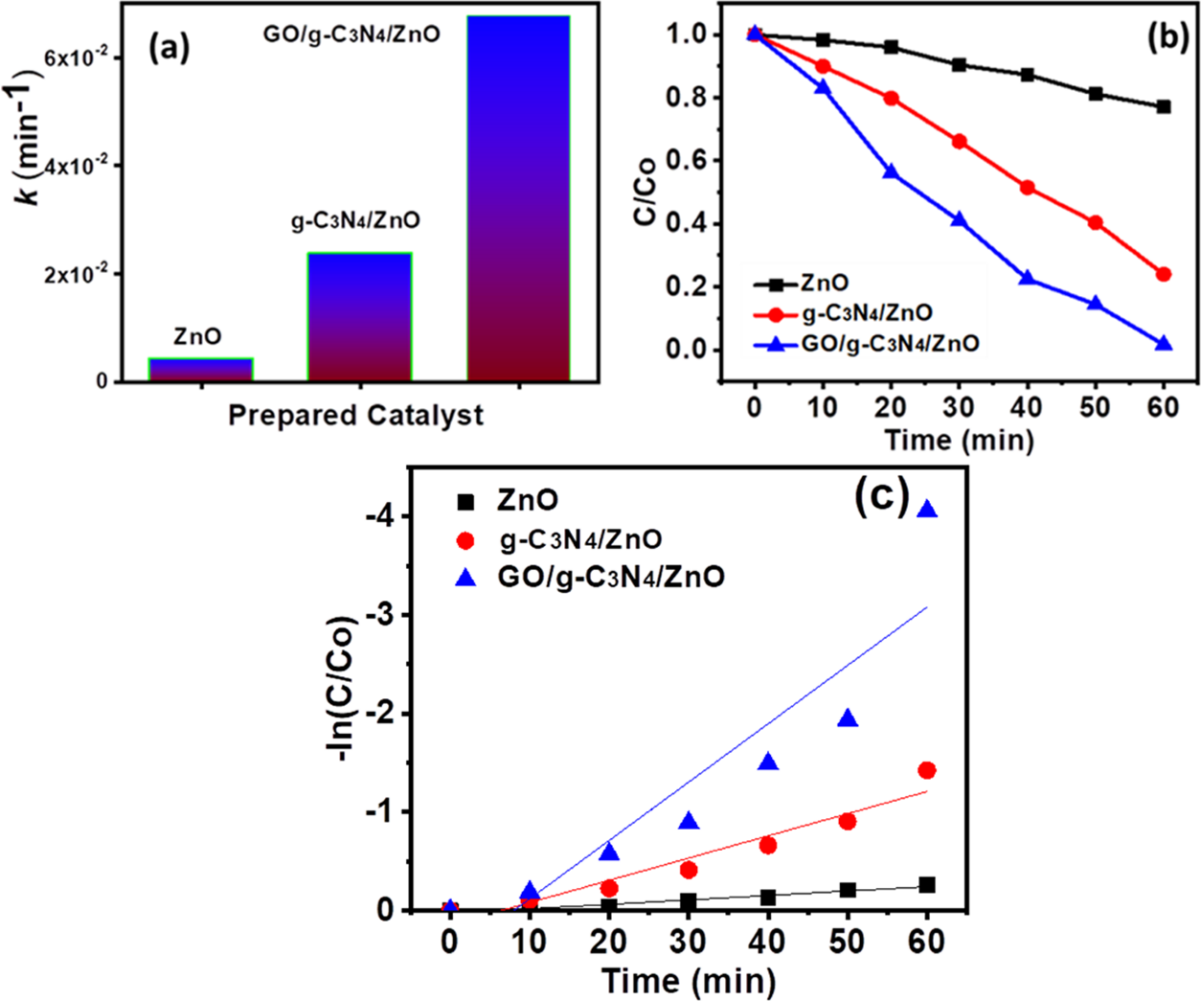
Degradation kinetics (a), apparent rate constant (b), and kinetics plot (c).

The rate constant and degradation kinetics were affected by adding different concentrations of prepared photocatalysts (Figure 6a–c). As we have seen in Figure 6b, when the photocatalyst was added, the degradation was increased with time.^51^ When reaction time was reached at 60 min, the degradation of MB was maximum with a mixture containing GO/g-C_3_N_4_/ZnO. The rate constant for GO/g-C_3_N_4_/ZnO was 0.006 after 60 min, 0.003 for g-C_3_N_4_/ZnO, and 0.001 for ZnO photocatalyst. It was noted that the apparent rate constant was the maximum for GO/g-C_3_N_4_/ZnO after 60 min.

In conclusion, the proposed photocatalytic degradation mechanism of MB by GO/g-C_3_N_4_/ZnO demonstrated an enhanced performance under visible light irradiation. The absorption of photons by g-C_3_N_4_ initiated electron acceleration from the valence band to the conduction band. Following which, these electrons transfer to the conduction band of ZnO, while holes migrated toward the valence band of g-C_3_N_4_. This charge carrier separation reduces recombination. Moreover, changes in the work functions of GO and ZnO facilitated electron acceptance from ZnO, further minimizing charge recombination.^52^ Consequently, the surplus charge carriers reached the surface of GO/g-C_3_N_4_/ZnO, where they plausibly engaged with water to generate hydroxyls and radicals.^53^ The ensuing interaction with MB led to an amplified degradation of the dye, highlighting the efficiency of the photocatalytic process.

### 3.6 UV–Visible Spectroscopy

**Figure 7:**
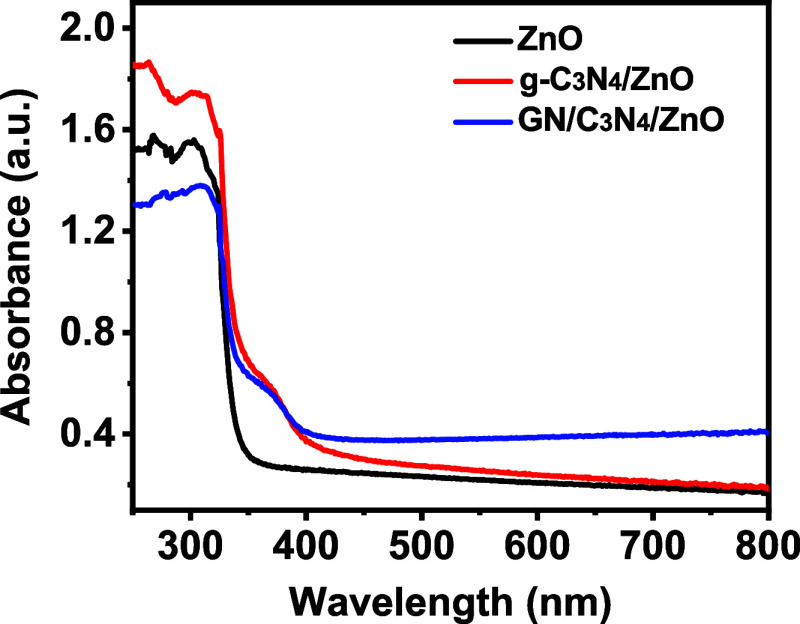
UV–vis spectra of prepared photocatalysts.

The photocatalytic degradability of ZnO, g-C_3_N_4_/ZnO, and GO/g-C_3_N_4_/ZnO composites was tested, and the results are shown in Figure 7. In Fenton’s photocatalytic-like oxidation process, active species were produced through the separation, migration, and coupling of photogenerated charge carriers. One of these elements is the photocatalyst’s capacity to absorb light, which impacts the Fenton-like oxidation process. To comprehend the absorption spectra property, the UV–vis absorption patterns of ZnO, g-C_3_N_4_/ZnO, and GO/g-C_3_N_4_/ZnO were investigated^37^ (Figure 7). Only ultraviolet light having a wavelength of 400 nm or less can break up by pure ZnO nanorods. However, the GO/g-C_3_N_4_ caused the absorption zone to grow after loading in the visible range.^37^ Additionally, the UV absorption edge of the photocatalyst exhibits a slight blue shift because of the size quantization effect brought on by the reduction in the ZnO nanorod size.

## 4 Conclusions

In conclusion, the addition of ZnO to g-C_3_N_4_/GO resulted in enhanced degradation of methylene blue through the photocatalytic processes during wastewater treatment. ZnO exhibited a 22.9% reduction in methylene blue concentration after 60 min of treatment. However, owing to its wide band gap that limits light absorption and its rapid electron–hole pair recombination rate during charge carrier conduction from the valence band to the conduction band, the catalytic activity of pure ZnO was somewhat diminished.

Specifically, the combination of ZnO and g-C_3_N_4_ led to an acceleration in methylene blue degradation, reaching 76.0%. Interestingly, the composite GO/g-C_3_N_4_/ZnO displayed the highest activity, with a notable 90% degradation of methylene blue achieved. In summary, the synthesized nanocomposite exhibited remarkable efficacy in removing methylene blue from polluted water. This prepared photocatalyst holds significant promise as an effective solution for eliminating toxic organic dyes from industrial wastewater discharges. It is therefore a valuable tool in addressing the environmental issue of tackling contaminated water discharges from industrial facilities.

## Supporting Information Available

XPS spectra and elemental analysis of prepared composite (PDF)
