# Supplementary material for: Enhancing the Photocatalytic Degradation of Methylene Blue with Graphene Oxide-Encapsulated g-C3N4/ZnO Ternary Composites
Source: ACS Omega. 2024 Mar 26;9(14):16187–95. doi: 10.1021/acsomega.3c10172 (PMC11007858; doi:10.1021/acsomega.3c10172)
Supplement: Supplementary file 1 — ao3c10172_si_001.pdf [file ao3c10172_si_001.pdf]

Supplementary Information

Enhancing Photocatalytic Degradation of Methylene Blue with Graphene Oxide Encapsulated *g*-C_3N_4/ZnO ternary Composites

Muhammad Hassan Shakoor[1], Muhammad Bilal Shakoor[2*], Asim Jilani[3], Toheed Ahmed[1], Muhammad Rizwan[4], Mohsin Raza Dustgeer[4], Javed Iqbal[3], Muhammad Zahid[5], Jean Wan Hong Yong[6**]

[1]Department of Chemistry, Riphah International University, Faisalabad Campus, Faisalabad, Pakistan

[2]College of Earth & Environmental Sciences, University of the Punjab, Lahore, Pakistan

[3]Center of Nanotechnology, King Abdulaziz University, 21589, Jeddah, Saudi Arabia

[4]Department of Environmental Sciences and Engineering, Government College University Faisalabad, Faisalabad, Pakistan.

[5]Department of Chemistry, University of Agriculture, Faisalabad 38000, Pakistan
[6]Department of Biosystems and Technology, Swedish University of Agricultural Sciences, 23456 Alnarp, Sweden

Corresponding author(s): *bilalshakoor88@gmail.com; **jean.yong@slu.se

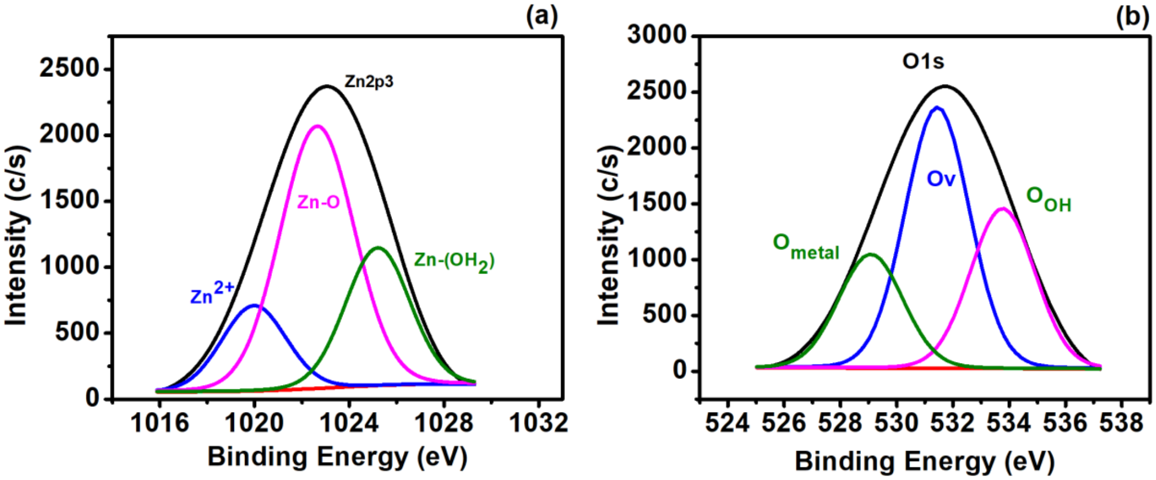

Figure S1: (a) Zn2p3 and (b) O1s spectrum of GO/g-C_3N_4/ZnO.

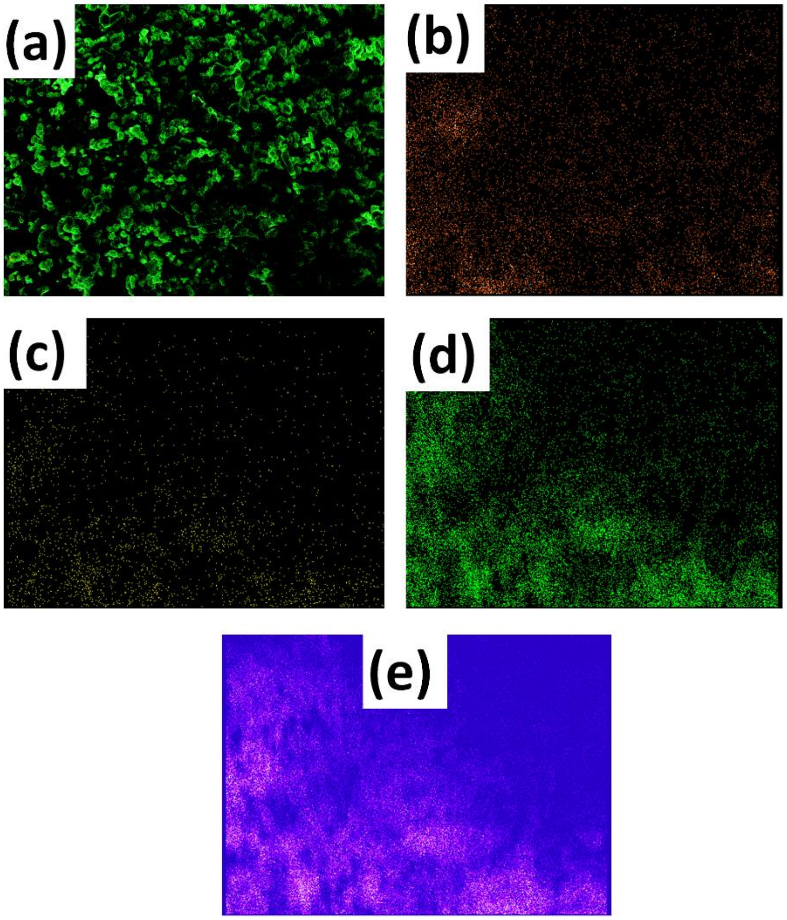

Figure S2: (a) compounded image (b-e) even dispersion of carbon, nitrogen, oxygen, and zinc of GO/g-C_3N_4/ZnO.
